# In Vitro Evaluation of Different Irrigation Protocols on Intracanal Smear Layer Removal in Teeth with or without Pre-Endodontic Proximal Wall Restoration

**DOI:** 10.3390/jcm9103325

**Published:** 2020-10-16

**Authors:** Naji Kharouf, Eugenio Pedullà, Giusy Rita Maria La Rosa, Frédéric Bukiet, Salvatore Sauro, Youssef Haikel, Davide Mancino

**Affiliations:** 1 Faculté de Chirurgie Dentaire, Université de Strasbourg, 8 rue Sainte Elisabeth, 67000 Strasbourg, France; youssef.haikel@unistra.fr (Y.H.); davidemancino@icloud.com (D.M.); 2 Institut National de la Santé et de la Recherche Médicale, Unité Mixte de Recherche 1121, 11 rue Humann, 67085 Strasbourg, France; 3 Department of General Surgery and Medical–Surgical Specialties, University of Catania, 95128 Catania, Italy; eugeniopedulla@gmail.com (E.P.); g_larosa92@live.it (G.R.M.L.R.); 4 Department of Endodontics, Aix Marseille Université, CNRS, ISM, Inst Movement Sci, 13385 Marseille, France; frederic.bukiet@univ-amu.fr; 5 Assistance Publique des Hôpitaux de Marseille, 13005 Marseille, France; 6 Departamento de Odontología, Facultad de Ciencias de la Salud, Universidad CEU-Cardenal Herrera, C/Del Pozo (s/n), Alfara del Patriarca, 46115 Valencia, Spain; salvatore.sauro@uchceu.es; 7 Department of Therapeutic Dentistry, I.M. Sechenov First Moscow State Medical University, 119146 Moscow, Russia

**Keywords:** active irrigation, pre-endodontic restoration, root canal, dentin, smear layer removal

## Abstract

To investigate the influence of pre-endodontic coronal wall restoration on smear layer removal during different root canal irrigation strategies, single-root premolars were prepared with a mesio-occlusal cavity. Half were left untreated (G1), while the mesial walls of the remaining half were reconstructed using a resin composite (G2). The specimens were divided into control (ctrl) groups, which used the conventional needle irrigation method, and four experimental subgroups according to irrigation strategy: Sonic activation using the Endoactivator, sonic activation using the EQ-S, mechanical activation using the XP-Endo finisher, and ultrasonic activation using the EndoUltra. Smear layer removal was assessed through SEM and the results were statistically analyzed. At the coronal and middle thirds, no significant difference (*p* > 0.05) was detected for G1 and G2, except for the control subgroups (G1ctrl vs G2 ctrl) at the middle third. At the apical third, the smear layer removal was significantly greater for G2 than G1. In G1, both at the middle and apical level, EQ-S and EndoUltra showed greater smear layer removal (*p* < 0.05) compared to the others G1 subgroups. In G2, at the apical level, the EQ-S and EndoUltra were the most effective in smear layer removal. Pre-endodontic coronal wall restoration may improve the smear layer removal during root canal irrigation.

## 1. Introduction

A successful root canal treatment depends on appropriate access cavity preparation, as well as proper shaping, appropriate cleaning, and accurate tridimensional sealing of the root canal system [1]. Several studies have demonstrated that substantial portions of the canal walls are not involved during mechanical instrumentation of the root canal [2,3]. This is due to round canal preparation, generated by most of the shaping instruments, that does not to correspond to the real root canal cross-section, which is often oval or irregular [2,4]. Moreover, it is well known that any mechanical instrumentation generates a smear layer, which is always characterized by organic and inorganic dental debrides [5,6]. In particular, the organic smear layer is constituted by mineralized dentin collagen mixed with residual predentin, pulp tissue, and microorganisms, along with their byproducts. This layer covering the dentin walls prevents sodium hypochlorite (NaOCl) penetration into dentinal tubules [7], where the presence of bacteria has been also demonstrated [8]. Thus, the cleaning and disinfection of the untreated root canal walls and the smear layer removal rely on specific irrigation protocol based on the use of a chelating agent, such as ethylenediaminetetraaceticacid (EDTA), and a proteolytic, antibacterial irrigant, such as NaOCl [9,10].

To enhance the efficacy of final intracanal irrigation, different activation/agitation techniques have been recommended after root canal shaping. For instance, sonic activation induces a mechanical oscillation, especially of the file tip, with a frequency ranging from 1 Hz to 6000 Hz [11], while the use of ultrasonic devices generates microstreaming along the file with a frequency ranging from 40,000 Hz to 45,000 Hz [12]. The Endoactivator (Dentsply Sirona, Ballaigues, Switzerland) is a further example of a battery-operated sonic cordless device, coupled with no-cutting polymer tips, which do not cut root dentin and are available in three sizes (15.02, 25.04, 35.04). It can be used at three different speeds, 10,000, 6000, and 2000 cycles per minute, generating a 160/190 Hz frequency [13] to improve the final irrigation protocol. Its design allows for the safe activation of intracanal irrigants, as well as irrigation inside curved root canals. More recently, the EQ-S (Meta Biomed, Chungcheongbuk-do, Korea), which is also based on sonic technology, was proposed. It is a cordless device that has two speeds, with a multidirectional movement and color-coded tips in three different sizes (15.02, 25.02, and 35.02) that can be used at two different speeds, 13,000 and 8000 cycles per minute, generating a 133/217 Hz frequency [14] used to boost endodontic intracanal irrigation in a safe way, as well as inside curved root canals. The EndoUltra (Micromega, Besançon, France) was the first cordless ultrasonic instrument generating a 40,000 Hz frequency. This device presents a rechargeable lithium ion battery and a single 15.02 stainless steel tip [13]. However, its rigid and nonflexible tip, especially in the curved root canals in contact with the dentinal walls, could create an unintentional intracanal ledge. In addition, the XP-Endo Finisher (FKG Dentaire SA, La Chaux-de-Fonds, Switzerland) has been proposed as a supplementary procedure to maximize the irrigation and disinfection effectiveness after root canal shaping. The XP-Endo Finisher is an ISO 25.00 instrument produced using NiTi MaxWire (Martensite-Austenite Electropolish-FleX, FKG). According to the manufacturer, the file is straight in its M-phase when cooled, and assumes a spoon shape when rotated and exposed to body temperature. It is suggested to be used at 800 rpm with irrigating solutions after root canal preparation to size #25 or larger.

Generally, teeth requiring endodontic treatment have extensive structural damage, especially after removing caries or old defective restorations [15]. Therefore, it has been advocated to place a pre-endodontic restoration in order to facilitate rubber dam placement, to easily define recognizable stable reference points for the rubber stops on the endodontic files, to reduce the risk of intraoperative marginal leakage, and to improve the temporization between two visits [16]. A pre-endodontic restoration can also act as reservoir for root canal irrigants, especially when employing activation/agitation techniques. However, while some practitioners often consider this clinical step as optional and time-consuming, most are concerned about pre-endodontic restoration’s benefits.

To the best of our knowledge, there is no study available that has investigated the impact of the presence of a pre-endodontic restoration on activation/agitation technique efficacy and cleaning ability.

Therefore, the aim of this study was to determine the influence of a pre-endodontic coronal wall restoration on smear layer removal in the coronal, middle, and apical thirds of a single root canal of the mandibular first premolars using five irrigation strategies. The null hypothesis tested was that the pre-endodontic restoration would have no effect on smear layer removal. The second null was that there would be no difference in smear layer removal between the irrigation strategies used in teeth with or without pre-endodontic restoration.

## 2. Experimental Section

### 2.1. Sample Selection

Sample size estimation was calculated with G*Power 3.1.9.2 software (Heinrich-Heine-Universität Düsseldorf, Düsseldorf, Germany). Ten 10 subgroups (including 2 controls) of 20 teeth each were finally formed in order to have 80% power and an alpha error probability of 0.05.

Three-hundred and seventy freshly mandibular first permanent premolars, extracted for orthodontic reason on patients aged between 18 and 24 years old, with fully formed apices and with a total length between 21 mm and 23 mm, were obtained under a patient-informed consent. The protocol was approved by the Ethics Committee, of Medical, Odontology School, and Strasbourg University Hospital (Protocol No. 2019-05). After debridement of the root surface, specimens were immersed in a 1% NaOCl solution at 4 °C for 24 h and then stored in saline solution.

Preselected teeth were first scanned with Cone Beam Computed Tomography (CBCT) and selected according to the following morphological criteria:

1. Single canal.
2. Canal with a long/short diameter ratio > 2 at 5 mm from the apex [17].
3. Canal with a total length of 14 ± 1 mm from canal orifice to apical foramen.
4. Primary root curvature in buccolingual and mesiodistal view ≤ 20° according to the Schneider method [18].
5. Main curvature radius ≥ 4 mm.

After selection, 210 teeth were finally included in the experimental design. Cusps were removed using silicon carbide grit papers (320 grit) mounted on a rotating polishing machine (Escil, Chassieu, France). This latter procedure was also used to obtain a constant reference point and to standardize the tooth length at 20 ± 0.1 mm.

### 2.2. Root Canal Preparation

**Figure 1:**
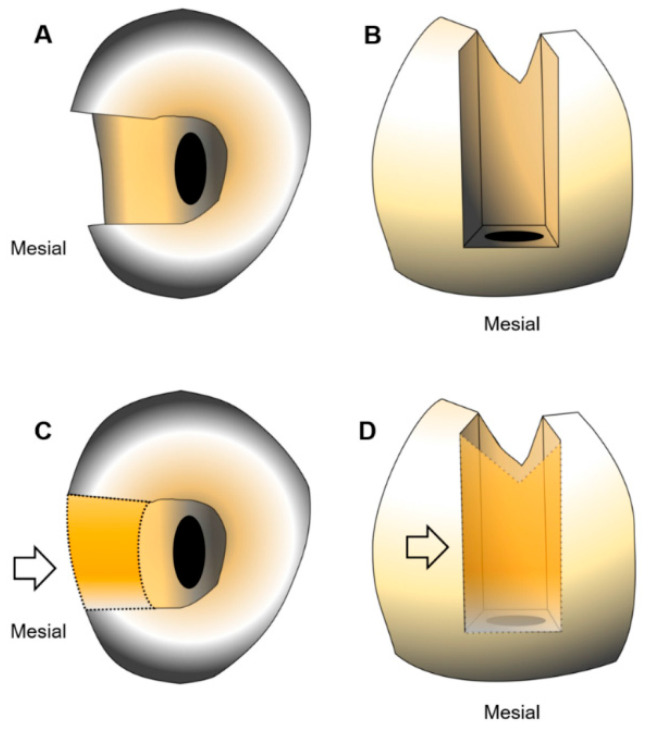
Representative picture of occlusal (**A**,**C**) and mesial (**B**,**D**) view of mandibular first premolars without (**A**,**B**) and with (**C**,**D**) pre-endodontic restoration. The orange dotted line indicates the mesial composite wall (arrows).

A single full-trained operator performed all the endodontic procedures in order to avoid interoperator variables. A calibrated mesial-occlusal (MO) cavity preparation was performed under a clinical microscope (Zumax Medical Co., Ltd., Suzhou, Jiangsu, China). The buccolingual cavity width was 4 mm and the proximal gingival margin was 1 mm above the CEJ. The samples were randomly divided in two equal groups (*n* = 100): G1, with a MO cavity and traditional access without a pre-endodontic restoration, and G2, with the mesial wall reconstructed using a universal adhesive (Iperbond Ultra, ITENA Clinical, Paris, France) and a composite resin restoration (Reflectys, ITENA Clinical, Paris, France) (G2) (Figure 1).

Canal scouting were performed in all specimens using a # 10 K-file. WL was established using a # 10 K-file under ×20 magnification by subtracting 0.5 mm from the length at which the tip of the instrument was visible at the apical foramen. Then, the apices were sealed with a cyanoacrylate glue in order to achieve a closed system [19,20]. The mandibular premolars were then immersed into a polyvinylsiloxane (PVS) impression materials up to cover the cemento-enamel junction.

Subsequently, an automated glide path enlargement was performed using ProGlider (Dentsply Sirona Endodontics, Ballaigues, Switzerland) powered by an endodontic engine (X-smart-IQ motor, Dentsply Sirona Endodontics, Ballaigues, Switzerland) in a continuous clockwise rotation at 300 rpm and 2.5 Ncm. Root canal shaping was performed using sequentially ProTaper Next X1 (tip size = 0.17 mm, taper = 0.04) and X2 (tip size = 0.25 mm, taper = 0.06) with the same parameters.

To ensure optimal cutting efficiency, new instruments were used to shape each canal (single use). During root canal shaping, after the use of each file, the root canal was irrigated with 1 mL of 6% NaOCl for (35 ± 5) s, for a total of 3 mL of NaOCl, using a 5 mL syringe (Coltene/Whaledent, Altstatten, Switzerland) with 31-gauge Navitip needles (Ultradent Products, South Jordan, UT, USA). The needle was inserted 1 mm short of its binding point during instrumentation. The maximal penetration depth of the needle tip was 1 mm short of the working length.

### 2.3. Final Irrigation Protocol

The specimens in groups G1 and G2 were randomly divided into a control (*n* = 20) and four experimental subgroups (*n* = 20) according to the different irrigation strategies used in the experimental study, (total number of subgroups: G1ctrl, G1a, G1b, G1c, G1d; G2ctrl, G2a, G2b, G2c, G2d). For each subgroup, volume and irrigation time were standardized as follows: 2.5 mL of 0.9% NaCl over (90 ± 5) s, 5 mL of 17% EDTA solution over (120 ± 10) s, 2.5 mL of 0.9% NaCl over (90 ± 5) s, 5 mL of a 6% NaOCl over (120 ± 10) s, followed by a final rinse with 2.5 mL of 0.9% NaCl over (90 ± 5) s. The irrigants were continuously delivered, drop by drop, into the access cavity for all subgroups using a 5 mL syringe with a 31-gauge Navitip needle, except for the control subgroup, where irrigants were continuously delivered to the apical third of the root canal.

The control subgroup needle was passively introduced into the root canal, 1 mm short of the WL, with gentle “in-and-out” movements of 1–2 mm amplitude, at an approximate rate of (70 ± 5) strokes/min.

- Subgroup a: Sonic activation with the Endoactivator used in combination with the red tip (25/0.04) at 10,000 cycles/min (166 Hz). The tip was introduced 1 mm short of the WL and used with an “in-and-out” movement of 3–4 mm amplitude, at an approximate rate of (70 ± 5) strokes/min.
- Subgroup b: Sonic activation with the EQ-S cordless sonic endo irrigator, used in combination with a medium tip (25/0.02) at 13,000 cycles/min (217 Hz). The tip was introduced 1 mm short of the WL and used with an “in-and-out” movement like in subgroup a.
- Subgroup c: Activation with the rotary XP-Endo finisher. The instrument was used coupled with the smart-IQ motor at 800 rpm and 1 Ncm. It was inserted into the canal without rotation, 1 mm short of the WL. The irrigants were continuously delivered, drop by drop, into the access cavity during the activation, and rotation was triggered using an “in-and-out” movement of 6-8 mm amplitude [21], at an approximate rate of 30 strokes/min.
- Subgroup d: Ultrasonic activation with the EndoUltra cordless device used with the Ni-Ti tip (15/0.02, oscillating at 40 KHz). The tip was introduced 2 mm short of the WL and moved with an “in-and-out” movement like in subgroups a and b.

The tips were chosen as recommended by the manufacturers for the Endoactivator and EQ-S. For the Endoultra, only one tip was available (15/0.02).

The remaining ten teeth, five with pre-endodontic restoration and five teeth without pre-endodontic restoration, were used as control teeth after a shaping procedure without final irrigation to observe the smear layer formation at apical, middle, and coronal thirds.

### 2.4. Scanning Electron Microscope (SEM) Preparations and Observations

After irrigation procedures, the specimens were removed from the PVS impression material and sectioned by cutting two shallow longitudinal grooves (approximately 0.5 mm.) in the buccolingual direction. Extreme care was taken to ensure that the grooves followed the canal curvature and did not penetrate into the canal.

**Figure 2:**
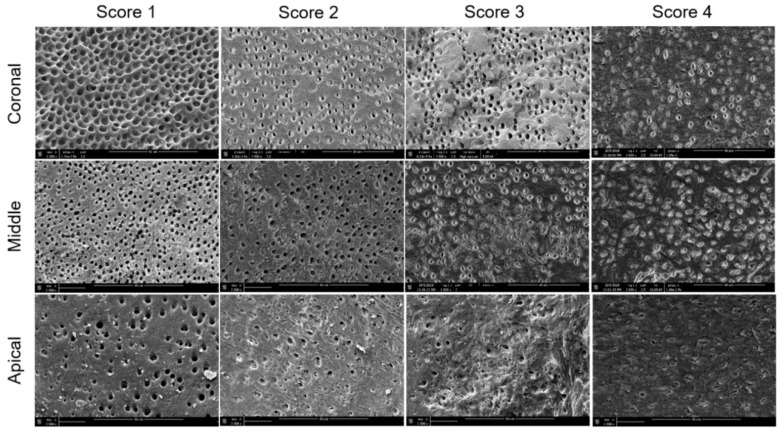
Representative scanning electron microscopy micrographs (×2000 magnification) of smear layer scores at the coronal, middle, and apical thirds.

All the specimens were then dehydrated in a graded series of ethanol solutions (50%, 70%, 90% and 100%, 3 min each) and sputter-coated with a gold-palladium alloy (20/80 weight %) using a HUMMER JR sputtering device (Technics, San Jose, CA, USA). The coated specimens were analyzed using the Quanta 250 FEG scanning electron microscope (FEI Company, Eindhoven, The Netherlands) at an electron-accelerating voltage of 5 kV to assess the smear layer removal at the coronal, middle, and apical thirds. One SEM micrograph showing the canal wall surface, in the area with the greatest amount of smear layer, at ×2000 magnification, was taken from the coronal, middle, and apical thirds of each half of the roots. A grading system based on the criteria proposed by Gutmann et al. [22] was applied to all micrographs to evaluate the smear layer removal (12 fields at magnification ×2000) (Figure 2).

The specimens were then scored as follows: Score 1, little or no smear layer, covering <25% of the specimen with tubules visible and patent; score 2, little to moderate or patchy amounts of smear layer, covering between 25% and 50% of the specimen with many tubules visible and patent; score 3, moderate amounts of scattered or aggregated smear layer, covering between 50% and 75% of the specimen with minimal to no tubules visible or patent; score 4, heavy smear layer covering over 75% of the specimen with no tubule orifices visible or patent. The SEM micrographs at ×2000 magnification, showing the canal wall surface of different subgroups at the coronal, middle and apical thirds, were coded for blinded notation, independently, by two experienced SEM examiners. When different scores were attributed by the two examiners, they reanalyzed the micrograph with a third examiner to reach an agreement.

### 2.5. Statistical Analysis

Cohen’s Kappa test was applied to verify the agreement between the two observers using Minitab software (Minitab^®^ 18.1, Minitab, Inc., Pennsylvania State University, PA, USA). The Shapiro–Wilk test was used to verify the normality of data within all subgroups in each group. However, normality was never verified. Thus, the Kruskal–Wallis test (One-Way Analysis of Variance on Ranks) was used including a multiple comparison procedure (Tukey post-hoc test). Data analyses were performed with Sigma Plot (11.2, Systat Software, Inc., San Jose, CA, USA). A significance level at *α* = 0.05 was adopted.

## 3. Results

**Figure 3:**
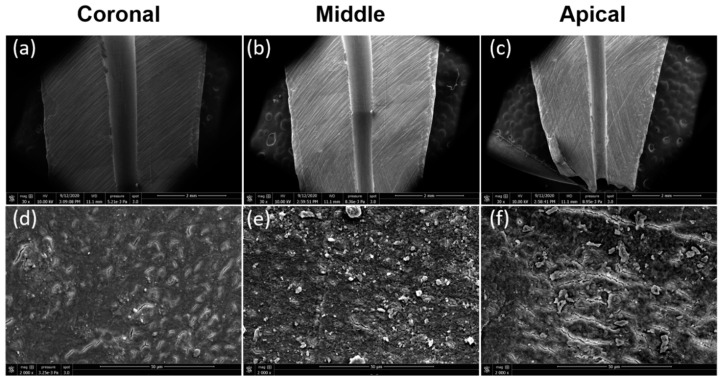
Representative scanning electron microscopy micrographs of longitudinal sectioned root surfaces after the shaping procedure. (**a**) Coronal third (×30 magnification); (**b**) middle third (×30 magnification); (**c**) apical third (×30 magnification); (**d**) coronal third (×2000 magnification); (**e**) middle third (×2000 magnification); (**f**) apical third (×2000 magnification).

**Figure 4:**
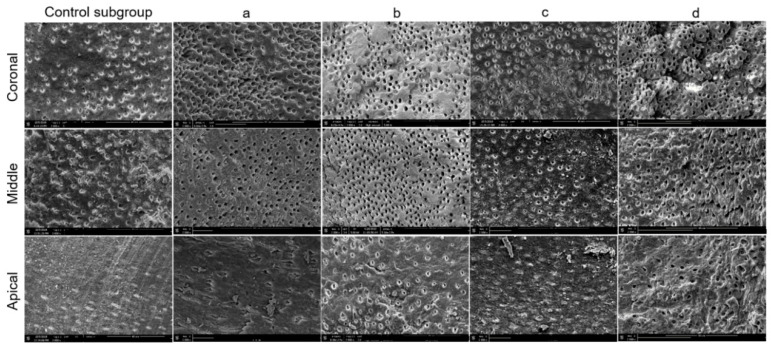
Representative scanning electron microscopy micrographs (×2000 magnification) of the coronal, middle, and apical thirds in samples from G1 (without pre-endodontic restoration). Five subgroups studied: (Control subgroup) Syringe and needles; (**a**) Endoactivator; (**b**) EQ-S; (**c**) XP-Endo finisher; (**d**) EndoUltra cordless.

**Figure 5:**
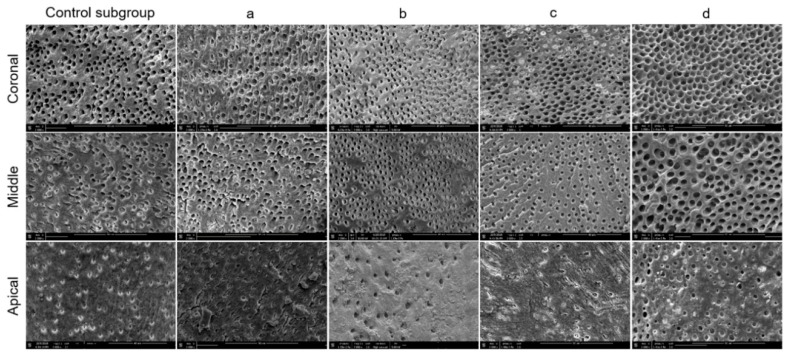
Representative scanning electron microscopy micrographs (×2000 magnification) of the coronal, middle, and apical thirds in samples from G2 (with pre-endodontic restoration). Five subgroups studied: (Control subgroup) Syringe and needles; (**a**) Endoactivator; (**b**) EQ-S; (**c**) XP-Endo finisher; (**d**) EndoUltra cordless.

The Cohen’s kappa value for interobserver agreement of all groups and subgroups was 0.89. The normality test (Shapiro–Wilk) failed for all data. Hence, the Kruskal–Wallis test was used to compare the data. The five control teeth with pre-endodontic restorations and the five control teeth without pre-endodontic restorations, examined after shaping procedures, indifferently showed a smear layer covering 100% of dentinal tubules at the three levels investigated (Figure 3). The results of SEM analysis for smear layer after the final irrigation protocol, for each group and subgroup, are summarized in Figure 4 and Figure 5.

**Figure 6:**
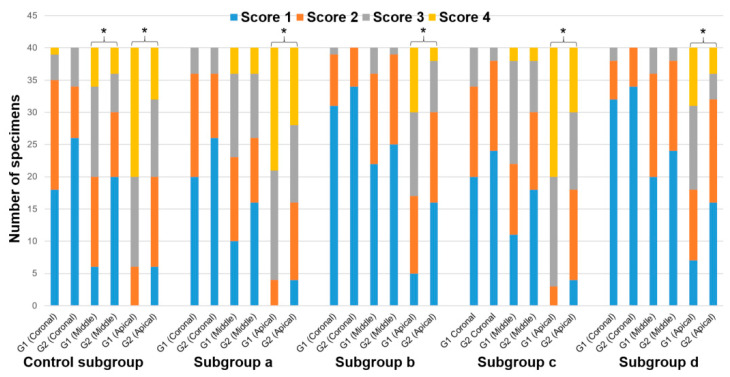
Number of specimens registered for each score of smear layer removal in the different root canal thirds and for each subgroup in groups 1 and 2 (* *p* < 0.05).

The scores and significant differences among groups (1 and 2) and subgroups are also summarized in Table 1 and Table 2, respectively, and in Figure 6.

In the coronal third, no significant difference (*p* > 0.05) was observed regarding the amount of smear layer removal, between the group G1 and group G2. In the middle third, no significant difference (*p* > 0.05) was found in any experimental subgroups between G1 and G2, except for the control subgroup (*p* < 0.05). In the middle third, the amount of smear layer was significantly lower for the control subgroup with pre-endodontic restoration (G2ctrl) in comparison to the control subgroup without pre-endodontic restoration (G1ctrl). In the apical third, for each subgroup, the amount of smear layer was significantly lower in the group with pre-endodontic restoration (G2) than in the group without pre-endodontic restoration (G1) (*p* < 0.05).

In the group without pre-endodontic restoration (G1), no significant statistical difference (*p* > 0.05) was seen in the coronal third of the root between the subgroups, whereas, for the middle and apical thirds, G1b (EQ-S) and G1d (EndoUltra cordless) presented a significantly lower amount of smear layer (*p* < 0.05) than the others G1 subgroups, with no difference between G1b and G1d, or between the other three remaining subgroups (*p* > 0.05).

In the group with pre-endodontic restoration (G2), no significant difference (*p* > 0.05) was found for coronal and middle thirds between the subgroups. In the apical third, subgroups G2b (EQ-S) and G2d (EndoUltra cordless) showed a significantly lower amount of smear layer (*p* < 0.05) than the other G2 subgroups. No difference was observed between G2b and G2d. Likewise, no difference was attained between the three remaining subgroups (*p* > 0.05).

For each subgroup without pre-endodontic restoration (G1), the amount of smear layer was significantly lower (*p* < 0.05) in the coronal and middle thirds than in the apical third. However, in the control subgroup and in G1a (Endoactivator), the coronal third showed a significant lower amount of smear layer than the middle third, while, in the remaining three subgroups (G1b, c, and d), no statistical difference was found between the middle and coronal thirds (*p* > 0.05). Regarding the specimens with pre-endodontic restoration (G2), the amount of smear layer was significantly lower in the coronal third than in the apical third of each subgroup (*p* < 0.05). However, in G2a (Endoactivator), the coronal third showed a significant lower (*p* < 0.05) amount of smear layer than the middle third, whereas, in the other four subgroups, the coronal third showed no significant difference in comparison to the middle third (*p* > 0.05). No statistical difference was found between the middle and apical thirds in groups G2b (EQ-S) and G2d (EndoUltra cordless) (*p* > 0.05).

## 4. Discussion

This study is the first work assessing the influence of pre-endodontic restoration on smear layer removal in the coronal, middle, and apical thirds of the root canal when using different irrigation strategies. In this study, the use of CBCT for the selection of the dental specimens permitted us to reduce the lack of homogeneity among the groups and subgroups [19]. Moreover, in order to standardize the irrigation procedure, each subgroup was irrigated under identical conditions using the same volume and flow rate. The only variables were represented by the activation technique, the taper, and the diameter of the tip used in combination with the different devices tested. The tip of each device tested was chosen according to the manufactured instructions. With regard to the Endoactivator tips, a previous study showed that there was no statistically significant difference between the 15/0.02 and 25/0.04 tips [23].

In the present study, a scoring system based on the criteria proposed by Gutmann et al. [22] was applied to all SEM micrographs to evaluate the amount of smear layer at the coronal, middle, and apical thirds. However, to minimize the limitations of the SEM analysis, which allows us to evaluate small areas of the canal walls, each third of the root canal was fully scanned under SEM, and the area with the greatest amount of smear layer was photographed and further analyzed, as recommended in a previous study [7,24].

With regard to the control specimens examined after shaping procedures, all showed a smear layer covering 100% of dentinal tubules at the three levels investigated. Therefore, such an outcome indicates that the areas observed were fully covered with smear layer before the cleaning procedures (Figure 3). As far as the samples analyzed after the final irrigation protocols, at the coronal level, regardless of the activation system used, no significant difference was detected for smear layer removal between the two groups tested in this study, with and without pre-endodontic restoration.

Likewise, at the middle level, except for the control subgroup, which was irrigated using the conventional needle irrigation method, no significant difference was observed on the smear layer removal between the two groups tested, with and without pre-endodontic restoration. This might be explained by the fact that a proper renewal of root canal irrigants in the coronal and middle thirds can be achieved regardless of the irrigation technique, as well as the presence/absence of a pre-endodontic restoration. This may be also confirmed by the fact that, in the group without pre-endodontic restoration (G1), the experimental subgroups already displayed relatively clean root canal walls (scores 1 and 2) at the middle level at a percentage between 52.5% and 90%.

At the apical level, G2 (with pre-endodontic restoration) presented a significantly lower amount of smear layer than G1 (without pre-endodontic restoration) for each subgroup evaluated. Thus, the first null hypothesis—that pre-endodontic restoration would have no effect on smear layer removal—can be rejected. Therefore, the presence of a pre-endodontic restoration may improve the cleaning ability of the irrigation protocol in the apical third, which is the less accessible area. Indeed, the pre-endodontic restoration acts as a reservoir, continuously maintaining a substantial volume of irrigant into the access cavity. This may enhance fluid dynamics, especially the crown-down streaming of root canal irrigants [25,26] during activation and the possibility to have fresh irrigants at the apical third. At the middle level, concerning the subgroups irrigated using the conventional needle irrigation method, a significantly lower amount of smear layer was found in G2ctrl (control) rather than in G1ctrl. This finding could be due to the fact that G1ctrl showed the “worst” results compared to the other subgroups without pre-endodontic restoration (G1).

In G1 (without pre-endodontic restoration), no statistical difference occurred at the coronal level on the smear layer removal between the different subgroups. However, at middle and apical level, subgroup (b), sonically activated with EQ-S (G1b), and subgroup (d), ultrasonically activated (G1d) with EndoUltra, showed a lower amount of smear layer compared to the other three subgroups. The absence of pre-endodontic restoration in G1 might decrease the volume of irrigant penetrating the canal and reaching the middle third and apical third. Therefore, irrigant penetration in such areas might be more influenced by the efficacy of the activation/agitation technique.

These latter results, concerning the ultrasonic agitation, are in accordance with those reported in previous studies [27,28,29], which showed how ultrasonic activation can enhance smear layer removal. However, to the best of our knowledge, no data exists in the literature regarding sonic activation with EQ-S. Regarding the Endoactivator device, the results are in agreement with those reported by Uroz-Torres et al. [30], who showed that this agitation technique did not enhance smear layer removal. However, it must be pointed out that other studies showed that the Endoactivator resulted in more smear layer removal than a conventional syringe irrigation [31,32]. Nevertheless, the different results might be attributed to the different shaping protocol, apical enlargement, and specimen selection, as well as to the different volume and flow rate of the irrigants.

Interestingly, G1b, where the irrigants were sonically activated with EQ-S, showed an amount of smear layer significantly lower than G1a, where the irrigants were sonically activated with Endoactivator. This difference might be attributed to the different features of each device, such as the oscillation frequency, the different pattern of movement, and the diameter/taper/composition of the tip. However, if we want to standardize the comparison between the Endoactivator and EQ-S, we would have had to also use a 25/02 tip for the Endoactivator. However, this tip is not commercialized. On the other hand, as mentioned above, concerning the 15/02 tip and 25/04 tip of the Endoactivator, no difference existed in amplitude, oscillatory pattern, or wall contact. Therefore, it could be hypothesized that the lower oscillation frequency of the Endoactivator and the different composition of the tip may decrease the streaming of irrigants.

Our results regarding G1c, where the irrigants were mechanically activated using the XP-Endo Finisher, are similar to the those reported by a previous study, which showed that such a file had no enhancing effect on smear layer removal [33]. However, our results are in partial disagreement with another study, which showed that XP-Endo Finisher activation had an improvement effect on smear layer removal when compared to the group irrigated using conventional needle irrigation method and that there was no significant difference when XP-Endo Finisher activation was compared to sonic activation using the Endoactivator in combination with a red tip (25/0.04) [34]. Other investigations showed that XP-Finisher agitation was as effective as ultrasonic agitation in smear layer removal [35,36]. Nevertheless, these different results might be attributed to the different shaping protocol, apical enlargement, and specimens’ selection, as well as to the different volume and flow rate of the irrigants.

Overall, in the group without pre-endodontic restoration (G1), none of the investigated irrigation strategies permitted us to properly remove the smear layer, with only 10–45% of the samples showing clean root canal walls (scores 1 and 2) at the apical level. These findings are in agreement with previous studies [29,37], where the teeth were instead decoronated before the final irrigation protocol.

In each subgroup analyzed without pre-endodontic restoration (G1), the amount of the smear layer was significantly lower in the coronal and middle thirds than in the apical third, as found in previous studies [7,24,28,37]. However, concerning the subgroup irrigated using the conventional needle irrigation method (G1ctrl) and the subgroup where the irrigants were sonically activated using Endoactivator (G1a), the coronal third showed a statistically lower amount of smear layer than the middle third, while in the remaining three subgroups, no significant difference was found between the middle and coronal thirds. These findings are in partial agreement with those of Urban et al. [28], who showed that smear layer removal, as well as canal decontamination, decreased from coronal to apical third.

In the group with pre-endodontic restoration (G2), no significant difference was detected in the coronal and middle thirds in terms of smear layer removal between any subgroups. It could be hypothesized that the presence of the pre-endodontic restoration might decrease the impact of the irrigation technique, because in G2, unlike in G1, even at the middle third, no statistical difference was found in terms of smear layer removal between the different subgroups. At the apical level, the subgroup where the irrigants were sonically activated with EQ-S (G2b) and the subgroup where the irrigants were ultrasonically activated with EndoUltra (G2d) showed a lower amount of smear layer compared to the other three subgroups.

Moreover, concerning these two subgroups, in the first one, where the irrigants were sonically activated with EQ-S (G2b), and the second one, where the irrigants were ultrasonically activated with EndoUltra (G2d), it was possible to remove the smear layer in 75–85% of the specimens, showing clean root canal walls at the apical level (scores 1 and 2). Thus, the second null hypothesis—that there would be no difference in smear layer removal between the irrigation strategies used in teeth with or without pre-endodontic restoration—can be rejected.

Concerning the group with pre-endodontic restoration (G2), the amount of the smear layer was significantly lower in the coronal third compared to in the apical third for each subgroup analyzed. Regarding the subgroup irrigated using the conventional needle irrigation method (G2ctrl), the subgroup where the irrigants were sonically activated using Endoactivator (G2a), and the subgroup where the irrigants were mechanically activated using the XP-Endo Finisher (G2c), the amount of the smear layer observed was significantly lower in the middle third than in the apical third. Whereas, in the subgroup where the irrigants were sonically activated using EQ-S (G2b) and in the subgroup where the irrigants were ultrasonically activated using EndoUltra (G2d), no significant difference was seen between the middle and the apical thirds on the smear layer removal. These findings could be due to a combination of different factors, such as the presence of pre-endodontic restoration, the continuous irrigation flow and the power of the oscillation frequency enhancing the renewal of irrigants, and the contact time between the latter and canal walls.

## 5. Conclusions

Within the limitations of this study, pre-endodontic restoration improved root canal irrigation procedure to remove the smear layer, especially in the apical third. Further studies are needed to confirm the findings of the present investigation, which tends to recommend pre-endodontic restoration placement prior to root canal treatment. Regarding the activation techniques, this work also showed that sonic activation with EQ-S and ultrasonic activation with EndoUltra cordless devices may provide a valid approach to achieve superior smear layer removal in the overall root canal length.

## Figures and Tables

**Table 1:** Number of samples registered for each score of smear layer removal in the different root canal thirds for the different irrigation techniques of G1 (without pre-endodontic restoration). Abbreviations in the last column and last row indicate significant differences between the different subgroups and/or thirds (*p* < 0.05); Co: Coronal, Mi: Middle, Ap: Apical; Subgroups: ctrl, a-d.

Canal Third	Score	G1 (Ctrl)	G1 (a)	G1 (b)	G1 (c)	G1 (d)	*p* Value < 0.05
**Coronal** **(Co)**	1	18	20	31	20	32	No significant differences
	2	17	16	8	14	6	
	3	4	4	1	6	2	
	4	1	0	0	0	0	
**Middle** **(Mi)**	1	6	10	22	11	20	Ctrl-b; Ctrl-d a-b; a-d c-b; c-d
	2	14	13	14	11	16	
	3	14	13	4	16	4	
	4	6	4	0	2	0	
**Apical** **(Ap)**	1	0	0	5	0	7	Ctrl-b; Ctrl-d a-b; a-d c-b; c-d
	2	6	4	12	3	11	
	3	14	17	13	17	13	
	4	20	19	10	20	9	
***p* value < 0.05**		Co-Mi Co-Ap Mi-Ap	Co-Mi Co-Ap Mi-Ap	Co-Ap Mi-Ap	Co-Ap Mi-Ap	Co-Ap Mi-Ap	

**Table 2:** Number of samples registered for each score of smear layer removal in the different root canal thirds for the different irrigation techniques of G2 (with pre-endodontic restoration). Abbreviations in the last column and last row indicate significant differences between the different subgroups and/or thirds (*p* < 0.05); Co: Coronal, Mi: Middle, Ap: Apical; Subgroups: ctrl, a-d.

Canal Third	Score	G2(Ctrl)	G2(a)	G2 (b)	G2 (c)	G2 (d)	*p* Value < 0.05
**Coronal** **(Co)**	1	26	26	34	24	34	No significant differences
	2	8	10	6	14	6	
	3	6	4	0	2	0	
	4	0	0	0	0	0	
**Middle** **(Mi)**	1	20	16	25	18	24	No significant differences
	2	10	10	14	12	14	
	3	6	10	1	8	2	
	4	4	4	0	2	0	
**Apical** **(Ap)**	1	6	4	16	4	16	Ctrl-b; Ctrl-d a-b; a-d c-b; c-d
	2	14	12	14	14	16	
	3	12	12	8	12	4	
	4	8	12	2	10	4	
***p* value < 0.05**		Co-Ap Mi-Ap	Co-Mi Co-Ap Mi-Ap	Co-Ap	Co-Ap Mi-Ap	Co-Ap	

## References

[1] Haapasalo M., Endal U., Zandi H., Coil J.M. (2005). Eradication of endodontic infection by instrumentation and irrigation solutions. Endod. Top..

[2] Ricucci D., Siqueira J.F. (2010). Fate of the tissue in lateral canals and apical ramifications in response to pathologic conditions and treatment procedures. J. Endod..

[3] Gutarts R., Nusstein J., Reader A., Beck M. (2005). In vivo debridement efficacy of ultrasonic irrigation following hand-rotary instrumentation in human mandibular molars. J. Endod..

[4] Passalidou S., Calberson F., De Bruyne M., De Moor R., Meire M.A. (2018). Debris Removal from the Mesial Root Canal System of Mandibular Molars with Laser-activated Irrigation. J. Endod..

[5] McComb D., Smith D.C. (1975). A preliminary scanning electron microscopic study of root canals after endodontic procedures. J. Endod..

[6] Torabinejad M., Handysides R., Khademi A.A., Bakland L.K. (2002). Clinical implications of the smear layer in endodontics: A review. Oral Surg. Oral Med. Oral Pathol. Oral Radiol..

[7] Tonini R., Giovarruscio M., Gorni F., Ionescu A., Brambilla E., Mikhailovna I.M., Luzi A., Maciel Pires P., Sauro S. (2020). In Vitro Evaluation of Antibacterial Properties and Smear Layer Removal/Sealer Penetration of a Novel Silver-Citrate Root Canal Irrigant. Materials (Basel).

[8] Kokkas A.B., Boutsioukis A.C., Vassiliadis L.P., Stavrianos C.K. (2004). The influence of the smear layer on dentinal tubule penetration depth by three different root canal sealers: An in vitro study. J. Endod..

[9] Trevisan L., Huerta I.R., Michelon C., Bello M.D.C., Pillar R., Souza Bier C.A. (2017). The Efficacy of Passive Ultrasonic Activation of Organic Solvents on Dissolving Two Root Canal Sealers. Iran. Endod. J..

[10] Goldman M., Goldman L.B., Cavaleri R., Bogis J., Sun Lin P. (1982). The efficacy of several endodontic irrigating solutions: A scanning electron microscopic study: Part 2. J. Endod..

[11] Mancini M., Cerroni L., Iorio L., Armellin E., Conte G., Cianconi L. (2013). Smear layer removal and canal cleanliness using different irrigation systems (EndoActivator, EndoVac, and passive ultrasonic irrigation): Field emission scanning electron microscopic evaluation in an in vitro study. J. Endod..

[12] de Gregorio C., Estevez R., Cisneros R., Heilborn C., Cohenca N. (2009). Effect of EDTA, sonic, and ultrasonic activation on the penetration of sodium hypochlorite into simulated lateral canals: An in vitro study. J. Endod..

[13] Pedullà E., Genovese C., Messina R., La Rosa G.R.M., Corsentino G., Rapisarda S., Arias-Moliz M.T., Tempera G., Grandini S. (2019). Antimicrobial efficacy of cordless sonic or ultrasonic devices on Enterococcus faecalis-infected root canals. J. Investig. Clin. Dent..

[14] Erdt O. EQ-S Cordless Root Canal Irrigant Activator. Initial Insight. August 2019. https://dentaladvisorblog.com/eq-s-cordless-root-canal-irrigant-activator/.

[15] Plotino G., Grande N.M., Isufi A., Ioppolo P., Pedullà E., Bedini R., Gambarini G., Testarelli L. (2017). Fracture Strength of Endodontically Treated Teeth with Different Access Cavity Designs. J. Endod..

[16] Castellucci A. (2006). Endodontics.

[17] Wu M.K., R’oris A., Barkis D., Wesselink P.R. (2000). Prevalence and extent of long oval canals in the apical third. Oral Surg. Oral Med. Oral Pathol. Oral Radiol..

[18] Schneider S.W. (1971). A comparison of canal preparations in straight and curved root canals. Oral Surg. Oral Med. Oral Pathol..

[19] Mancino D., Kharouf N., Hemmerlé J., Haïkel Y. (2019). Microscopic and Chemical Assessments of the Filling Ability in Oval-Shaped Root Canals Using Two Different Carrier-Based Filling Techniques. Eur. J. Dent..

[20] Kharouf N., Arntz Y., Eid A., Zghal J., Sauro S., Haikel Y., Mancino D. (2020). Physicochemical and Antibacterial Properties of Novel, Premixed Calcium Silicate-Based Sealer Compared to Powder–Liquid Bioceramic Sealer. J. Clin. Med..

[21] Bao P., Shen Y., Lin J., Haapasalo M. (2017). In Vitro Efficacy of XP-endo Finisher with 2 Different Protocols on Biofilm Removal from Apical Root Canals. J. Endod..

[22] Gutmann J.L., Saunders W.P., Nguyen L., Guo I.Y., Saunders E.M. (1994). Ultrasonic root-end preparation. Part 1. SEM analysis. Int. Endod. J..

[23] Jiang L.M., Verhaagen B., Versluis M., van der Sluis L.W. (2010). Evaluation of a sonic device designed to activate irrigant in the root canal. J. Endod..

[24] Plotino G., Özyürek T., Grande N.M., Gündoğar M. (2019). Influence of size and taper of basic root canal preparation on root canal cleanliness: A scanning electron microscopy study. Int. Endod. J..

[25] Türkün M., Cengiz T. (1997). The effects of sodium hypochlorite and calcium hydroxide on tissue dissolution and root canal cleanliness. Int. Endod. J..

[26] Loroño G., Zaldivar J.R., Arias A., Cisneros R., Dorado S., Jimenez-Octavio J.R. (2020). Positive and negative pressure irrigation in oval root canals with apical ramifications: A computational fluid dynamics evaluation in micro-CT scanned real teeth. Int. Endod. J..

[27] Alaçam T. (1987). Scanning electron microscope study comparing the efficacy of endodontic irrigating systems. Int. Endod. J..

[28] Urban K., Donnermeyer D., Schäfer E., Bürklein S. (2017). Canal cleanliness using different irrigation activation systems: A SEM evaluation. Clin. Oral. Investig..

[29] Karade P., Johnson A., Baeten J., Chopade R., Hoshing U. (2018). Smear Layer Removal Efficacy Using EndoActivator and EndoUltra Activation Systems: An Ex Vivo SEM Analysis. Compend. Contin. Educ. Dent..

[30] Uroz-Torres D., González-Rodríguez M.P., Ferrer-Luque C.M. (2010). Effectiveness of the EndoActivator System in removing the smear layer after root canal instrumentation. J. Endod..

[31] Caron G., Nham K., Bronnec F., Machtou P. (2010). Effectiveness of different final irrigant activation protocols on smear layer removal in curved canals. J. Endod..

[32] Blank-Gonçalves L.M., Nabeshima C.K., Martins G.H.R., Machado M.E. (2011). Qualitative analysis of the removal of the smear layer in the apical third of curved roots: Conventional irrigation versus activation systems. J. Endod..

[33] Azimian S., Bakhtiar H., Azimi S., Esnaashari E. (2019). In vitro effect of XP-Endo finisher on the amount of residual debris and smear layer on the root canal walls. Dent. Res. J. (Isfahan).

[34] Elnaghy A.M., Mandorah A., Elsaka S.E. (2017). Effectiveness of XP-endo Finisher, EndoActivator, and File agitation on debris and smear layer removal in curved root canals: A comparative study. Odontology.

[35] Leoni G.B., Versiani M.A., Silva-Sousa Y.T., Bruniera J.F.B., Pécora J.D., Sousa-Neto M.D. (2017). Ex vivo evaluation of four final irrigation protocols on the removal of hard-tissue debris from the mesial root canal system of mandibular first molars. Int. Endod. J..

[36] De-Deus G., Belladonna F.G., Zuolo A.S., Cavalcante D.M., Carvalhal J.C.A., Simões-Carvalho M., Souza E.M., Lopes R.T., Silva E.J.N.L. (2019). XP-endo Finisher R instrument optimizes the removal of root filling remnants in oval-shaped canals. Int. Endod. J..

[37] Rödig T., Döllmann S., Konietschke F., Drebenstedt S., Hülsmann M. (2010). Effectiveness of different irrigant agitation techniques on debris and smear layer removal in curved root canals: A scanning electron microscopy study. J. Endod..

